# Design Aspects of Additive Manufacturing at Microscale: A Review

**DOI:** 10.3390/mi13050775

**Published:** 2022-05-15

**Authors:** Nikolaos Rogkas, Christos Vakouftsis, Vasilios Spitas, Nikos D. Lagaros, Stelios K. Georgantzinos

**Affiliations:** 1 Laboratory of Machine Design, National Technical University of Athens, 9 Iroon Polytechniou, 15780 Zografou, Greece; nrogkas@mail.ntua.gr (N.R.); cvakouftsis@mail.ntua.gr (C.V.); vspitas@central.ntua.gr (V.S.); 2 Institute of Structural Analysis and Antiseismic Research, School of Civil Engineering, National Technical University of Athens, 9 Iroon Polytechniou, 15780 Zographou, Greece; nlagaros@central.ntua.gr; 3 Laboratory for Advanced Materials, Structures and Digitalization, Department of Aerospace Science and Technology, National and Kapodistrian University of Athens, Evripus Campus, 34400 Psachna, Greece

**Keywords:** additive manufacturing, microscale, design, materials, processes, applications

## Abstract

Additive manufacturing (AM) technology has been researched and developed for almost three decades. Microscale AM is one of the fastest-growing fields of research within the AM area. Considerable progress has been made in the development and commercialization of new and innovative microscale AM processes, as well as several practical applications in a variety of fields. However, there are still significant challenges that exist in terms of design, available materials, processes, and the ability to fabricate true three-dimensional structures and systems at a microscale. For instance, microscale AM fabrication technologies are associated with certain limitations and constraints due to the scale aspect, which may require the establishment and use of specialized design methodologies in order to overcome them. The aim of this paper is to review the main processes, materials, and applications of the current microscale AM technology, to present future research needs for this technology, and to discuss the need for the introduction of a design methodology. Thus, one of the primary concerns of the current paper is to present the design aspects describing the comparative advantages and AM limitations at the microscale, as well as the selection of processes and materials.

## 1. Introduction

Current advancements in design processes and procedures for additive manufacturing technology require the sufficient capturing of the scale aspect from a perspective that incorporates the evaluation of the overall dimensions (macro-aspect) and the local features (micro-aspect). This includes the different functionalities and/or physical laws that govern them, as well as their synergy, including individual features and macroscopic tolerances and sensitivity analysis. In order to take into account the scale aspect, modeling, analysis, and simulation tools, as well as manufacturing and experimental methods, have been employed that can treat micro- and nanoscale problems efficiently, treating them either as individual entities or combined [1,2,3] (multi-scale approach). The efficient capturing of the underlying physics (i.e., that governing physical laws), the level of material modeling, the manufacturing methods employed, and the metrological considerations for the evaluation of the product performance are of major importance during both the design and the manufacturing phase, which, at the microscale, suffers from a major dilemma. The above concerns have been heavily discussed and investigated over the past few years at the meso/macroscale [4,5] and, during the last two decades, at the nanoscale [6,7]. However, although the distinction between macro- and nanoscale tools is well established, the tools and methodologies of these scales can be applied and frequently find a use in research into microscale applications, by underutilizing or fitting these methodologies to various microscale aspects. Sometimes, this may lead to the treatment of microscale as a fuzzy boundary between meso and nanoscale, rendering both approaches valid and invalid at the same time without providing any clear distinction between them. For instance, the case of the contact between asperities in tribological applications can be considered as macro-, micro- or nanoscale, depending on the number of asperities that are in contact [8]. Nanoscale corresponds to single-asperity contact, while macroscale corresponds to contact between millions of asperities. However, microscale is represented as contact between a few asperities; thus, it is not accurately or quantitatively defined.

Design at the nanoscale follows the physics prevailing at the nanoscale level (i.e., hydrogen bonds, van der Waals forces, London forces, Pauli repulsions, etc.) along with the manufacturing considerations for scale-specific methodologies (i.e., laser ablation [9,10], electrochemical machining [11,12,13], photo-etching [14], chemical vapor decomposition [15] or physical vapor decomposition [16]) either for material removal (machining) or for material addition (additive manufacturing—AM). Macroscale design follows the laws of physics prevailing at the macroscale (i.e., classical continuum mechanics, flow dynamics, the isotropy of material properties, etc.) and conventional manufacturing methods, which are validated for the forming of small-scale structures (micromachining [17]) or the control of the texture (surface roughness) using superfinishing, lapping, etc.

Therefore, it is considered that the clear and concrete definition of microscale from a perspective that includes the analysis and simulation tools and the manufacturing methods, is a necessary step before proceeding to the design problem. The same applies to Additive Manufacturing (AM) which was originally used for the manufacturing of components in applications on a macro scale but, in recent years, this expanded to micro- and even nanoscale, either by creating new AM manufacturing processes or by adjusting the existing processes to fit smaller scales. Therefore, the tools and methodologies originally developed for macro-scale manufacturing were usually implemented, with adjuncts, to study and evaluate smaller scales. Moreover, AM, as a relatively new fabrication method that is completely different from the traditional subtractive manufacturing methods, poses a challenge in creating a design methodology fitted to its unique capabilities and strict limitations, to fully exploit its potential and revolutionize the manufacturing industry.

Today, the benefits of AM are well-established: design freedom, partial consolidation, no tooling being necessary, just-in-time inventory, faster production, easy accessibility, cost-effectiveness, tangible and creative designs, unlimited shapes and geometry, a variety of raw materials, less waste production, risk reduction, and others [18]. AM or three-dimensional printing (3DP) was developed in the 1980s as a rapid prototyping method and is now considered to be a manufacturing process in the same genre as conventional manufacturing processes. AM has generated an impact in all industry areas, including aerospace [19,20], automobile manufacture [21], construction [22], and medical and military applications [23]. It offers flexibility in the product design process and a considerable reduction in material consumption, as well as making product personalization affordable [24]. ASTM International categorizes AM processes into 7 groups [25]: i.e., material extrusion (e.g., fused deposition modeling—FDM), powder bed fusion, vat photopolymerization (stereolithography—SLA), material jetting (e.g., PolyJet), sheet lamination, directed energy deposition, and binder jetting, each typified by the principle according to which the resulting matter is formed.

Although AM techniques have progressed greatly at the macro-scale, many challenges remain to be addressed, mainly at micro- and lower scales. AM fabrication at these scales is associated with certain constraints, such as feature size limitations, the expansion of the range of materials used in order to include alternative ones (i.e., non-metallics, ceramics, composites, etc.), the improvement of surface quality and the minimization of porosity, as well as other geometrical defects. The rapid evolution of the current AM fabrication technologies, in addition to those newly introduced, does not give enough time for the introduction of a design methodology that addresses all the aforementioned limitations. Therefore, AM design methodologies need to involve and continuously adjust, based on the rapid evolution of AM technologies and processes.

This paper reviews the existing AM processes, their underlying techniques, commercial systems, and the materials used in AM fabrication at the microscale, together with applications in the production of microscale actuators, soft robotics, as well as biomedical and microfluidic devices. The primary concern is the presentation of design aspects describing the comparative advantages and AM limitations at the microscale, as well as the selection of processes and materials. The goal of this paper is to introduce the main design aspects of microscale AM, as described in the literature over the last five to ten years, and adumbrate a design methodology that is better fitted for microscale AM.

## 2. Technologies and Materials for AM at the Microscale

Additive manufacturing is a relatively new manufacturing method with increased popularity, aiming to expand the manufacturing capabilities of functional components. Among other applications, AM is used for the fabrication of products at micro- and nanoscale, with various degrees of geometrical and functional complexity and ever-increasing market infiltration into these areas. ISO/ASTM 52910-17 [25] is a standard that tries to set some guidelines regarding common industry practices in the field of AM at macro/meso scale; its extension to micro- or nanoscale is both an opportunity and a challenge. Although micro- and nanoscale AM is a relatively new field of application, macro-scale technologies are still of use to fabricate complex parts at these smaller scales. Many researchers, such as Vaezi et al., Paul et al., Behera et al., and Chizari et al. [26,27,28,29,30] proposed different classification categories for the application of these technologies at such scales, based on the production equipment used, the materials, the dimensions and the required tolerances of critical features, as well as other product attributes (i.e., intended use, texture, color, and strength). The most popular classification takes into consideration well-established macro-AM technologies, including 2D ink printing and other technologies fitted to the micro- and nanoscales. These can be divided into three main groups: macro or scalable additive manufacturing, 2D ink writing, or 3D direct writing processes, as well as hybrid ones.

### 2.1. Macro-AM Processes Fitted to Microscale Fabrication

The first group consists of traditional macro-AM processes fitted to micro- and nanoscale fabrication. Among them are the technologies widely used for macro-AM, such as Stereolithography, Selective Laser Sintering (SLS), Fused Deposition Modeling (FDM), Laminated Object Manufacturing (LOM), and inkjet printing processes. These scalable processes are adapted to microscale fabrication (micro-stereolithography or MSL, micro-laser sintering or MLS) but still face many difficulties and limitations. Modern micro-stereolithography (μ-SLA) pushes the resolution limits down to the sub-100 μm range with precision optics. The most common materials used are SL resins, hydrogels, biocompatible materials, and bioactive agents for a variety of bio-functional, implantable tissue-engineering applications, including nerve regeneration and guided angiogenesis [31,32,33,34,35,36]. Two-photon photopolymerization/lithography (TPL) is a photopolymerization-based technique that is primarily applied for the printing of polymer materials. Other materials are biocompatible and organic, mixing additives into the resist blend to generate composite structures, such as electrically conductive polymer microstructures loaded with carbon nanotubes [37,38,39,40,41,42,43,44]. Micro-SLS uses a laser to sinter small particles, consolidating powders in a layer-by-layer manner. The commercially available materials used in SLS come in powder form and include, but are not limited to, polymers such as polyamides (PA), polystyrenes (PS), thermoplastic elastomers (TPE), and polyaryletherketones (PAEK) [45,46,47]. Laminated object manufacturing is older and is slightly different from the known technologies and processes. LOM technology is based on the layer-by-layer fabrication of parts using sheets of various materials. Each sheet is cut into the desired geometry and used as a layer on top of the previous materials. The use of a binding substance ensures adhesion and creates the final functional component. Ceramics and metals, such as 316 L stainless steel, are among the most popular materials for LOM. Fused deposition modeling (FDM) is the most widely known and used AM process in macroscale fabrication. Nevertheless, it is fitted into producing components at the microscale as well. New developments help adapt to microscale needs and overcome limitations in terms of shape, resolution, and material usage. Polymers, metals, composites, and even biomaterials for tissue engineering are some of the most popular FDM materials.

### 2.2. Two-Dimensional Ink Writing Technologies

The second group of AM processes is based on 2D Ink writing technologies. These processes, traditionally used in the past for ink writing, are fitted for the fabrication of 3D microstructures. One of the most popular 2D-based methods is chemical vapor deposition (CVD). CVD creates a coating induced by a chemical reaction at the surface of a heated material. Laser chemical vapor deposition (LCVD) is a modified CVD process for the deposition of thin films. The two main categories of LCVD are photolytic LCVD and pyrolytic LCVD. In the first subcategory, the energy of the focused laser beam is absorbed by reagent gases, leading to the decomposition of gas molecules and the formation of a thin solid film on the substrate. In the second subcategory, the laser beam is focused on the locations to be deposited. This way, the temperature locally increases on the substrate until it reaches the threshold required. This leads to the deposition of a thin solid film on the substrate. Any material electroplated with nickel/chrome or stainless steel best supports PVD coatings. The most common chrome-plated materials are brass, zinc, steel, aluminum, and ABS plastic. Focused ion-beam direct writing (FIBDW) is another multi-material AM method that can use metallic, ceramic, and polymer inks for the fabrication of microscale structures. All inks must have specific rheological properties in order to be able to flow continuously through the printing nozzle and form a continuous filament, similar to that in FDM printing. This flow must be continuous, consistent, and able to create a discrete shape and form of layer [27,48,49,50]. Laser-induced forward transfer (LIFT) has been used to deposit a variety of materials, such as metals (Cu, Ag, Au, Pt, Cr, Al), semiconducting materials such as Ge and Se, oxide layers, nanocomposites, conductive polymers (PEDOT-PSS), biomaterials, and superconductors, among others. Donor materials with viscosities ranging from 10 to 100,000 cP have been printed with LIFT [51,52,53,54,55,56,57,58,59,60,61]. Another popular process, electrohydrodynamic (EHD) printing is a spray-based printing process that can pattern functional materials. EHD printing has been used to deposit metallic, carbon-based, ceramic, and polymer-based conductive materials, semiconducting nanoparticles (quantum dots), biomaterials, and molten metals on a wide range of substrates [62,63,64,65,66,67,68,69,70,71].

### 2.3. Hybrid Processes

The last category consists of methods combining additive and subtractive processes for micro-3D fabrication. Some typical methods are shape deposition modeling (SDM) and electrochemical fabrication (EFAB). SDM processes utilize additive and subtractive processes sequentially to produce 3D structures, but their use in micro-AM is limited. The EFAB process is based on the multilayer electrodeposition and planarization of at least two metals: one structural material and one sacrificial material. This process is capable of manufacturing microdevices with features as small as 20 μm and tolerances of ±2 μm. It is a popular method of manufacturing complex mechanisms without the need for assembly, which is favorable for medical devices. Common materials are Val-loy-120 (Ni–Co alloy), Edura-180 (electroplated Rh), and palladium [72].

From the above categorization, which is explored in more detail by the authors of [26], it can be deduced that the field of micro- and nano-AM both borrows existing practices from the mesoscale, expanding them into smaller sizes, and is based on either existing or new dedicated techniques at the micro- and nanoscales. The ability to control the geometry and tolerances at such a small scale is clearly one of the major issues, which, however, is at odds with the productivity of the more accurate yet slower dedicated techniques. Furthermore, the suitability of the proposed techniques for the desired material, size, accuracy, and productivity must be thoroughly assessed before the selection of the appropriate method.

## 3. AM Applications at the Microscale

Microscale AM processes have been employed in many technological fields for the fabrication of miniature devices. This paper addresses mainly recently published articles in the field of the design of microscale actuators and biomedical and microfluidic devices.

### 3.1. Αctuator Applications

In the field of micro and miniature actuators, microscale AM is indicated as a promising fabrication solution, and it promotes the production of micromachines with complex geometry using monolithic approaches, which would otherwise require a combination of advanced micro-subtractive manufacturing methods and, usually, assemblies with a large number of components. Recent review papers present and discuss the use of AM processes in the design and fabrication of microelectromechanical systems (MEMS) actuators, biohybrid actuators, and piezoelectric systems as relevant applications. In [73,74], the authors investigated the recent developments and achievements regarding the most widely used 3D printing technologies for MEMS fabrication and discussed their challenges and potential. Several papers [75,76] presented recent advancements in the field of small-scale soft robotics and actuators using AM, while other researchers [77,78] examined the application of 3D printing for the fabrication of piezoelectric actuators; finally, the authors of [79,80] discussed an approach regarding the fabrication of biohybrid actuators using AM.

**Figure 1:**
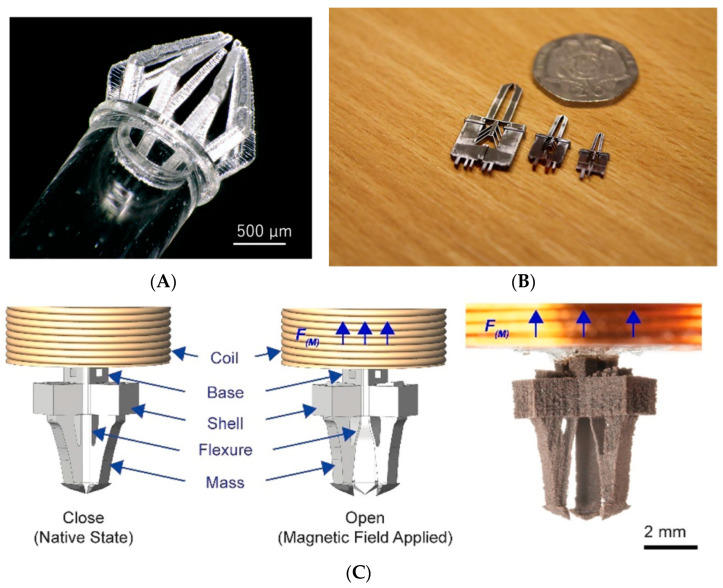
Examples of fabricated micro-grippers: (**A**) Micro-gripper for handling microstructure spheroids, mounted on a glass capillary. Reprinted with permission from Ref. [86]. Copyright 2020 MDPI. (**B**) Fabrication of a thermally actuated micro-gripper using a sputter-coating process for the deposition of metallic layers on polymer parts. Reprinted with permission from Ref. [88]. Copyright 2017 IOP Publishing. (**C**) Magnetically actuating micro-gripper for operation in air and water. Reprinted with permission from Ref. [82]. Copyright 2021 Elsevier.

The fabrication of micro-grippers is an indicative example revealing the importance of micro–AM. Accurate tip displacements, which are as small as 20 μm, are necessary for handling and pick-and-place, and the sterile handling of sensitive parts, which is a common procedure in the biology and health sector environments. The actuation principle may be piezoelectric, magnetic, or electrothermal. Shao et al. fabricated magnetically active 3D microstructures using a high-resolution micro-continuous liquid interface production process (μCLIP), combining 3D-printed centimeter-sized samples with sub-75 μm fine features [81]. The magnetic photopolymerizable resin that was used maintains high solid loading (30 wt % Fe_3_O_4_ nanoparticles), improves the surface properties by reducing the stair-like surface roughness, and accelerates the fabrication process. In another study [82], the authors used the same method (μCLIP) to fabricate a 3D printed magnetically driven triple-finger micro-gripper (Figure 1C), and tested its efficiency using a 300 μm diameter microsphere, both in air and in deionized water. The printing process involved the soaking of the part in acetone to remove the residual liquid resin (2 min), then its transfer into ethanol and ultrasonic cleaner (5 min), and finally, after drying (30 min), the specimens were post-cured in UV light of a 405 nm wavelength (10 min). Daniel et al. [83] fabricated a chevron-type electrothermal actuator, using the material extrusion-based manufacturing of a shape memory polymer composite. Using a resistivity of 1.8 Ωcm and an operational voltage as low as 3 V, they accomplished 100 μm tip displacement, which was computationally and experimentally investigated. Their main computational finding was that the grippers can be actuated quickly (3–5 s) with voltages as low as 5 V, but they recover slowly (60–100 s). Experimentally, higher voltages were required for actuation; a tip displacement of up to 77–117 µm was achieved in 5 s with an operational voltage of 17.5–19.5 V. In [84], Tyagi et al. used a custom-built syringe-based extrusion 3D printer to fabricate bilayer micro-actuators, driven by hydrogels, down to a size of 300 × (1000 ÷ 5000) µm^2^, with a minimum thickness of 30 µm. The printing resolution was 25 μm in the *x-y* plane; the rate of the lateral motion of the stage was ~2.5 mm/s and the air-dispensing pressure was 50–65 psi. The printing ink consisted of dissolved Hydromend D4 (hydrogel) in ethanol at a concentration of 20%. Lantada et al. [85] presented the development process of geometrically complex micro-vascular shape-memory polymer actuators by laser SLA, using a shape-memory epoxy that could change its shape as an effect of temperature increase. They presented two proof-of-concept applications: an active micro-claw with inner vasculatures of different cross-sections and an active spring with inner vasculatures of different cross-sections. In order to assess the effect of temperature on the closing of the gripper and the compression of the spring, they heated the prototypes with water flow (80 ℃) running through the micro-vasculatures. In [86], Kozaki et al. presented the design of a microgripper for handling spheroid microstructures, mounted on a glass capillary (Figure 1A). They used a top-down micro-stereolithography setup, based on a 405-nm blue laser developed in their previous study [87]. The photo-curable polymer used is a mixture of acrylate resin and a photopolymerization initiator, polymerization inhibitor, and blue light absorber (wavelength 405 nm). The mixture was mixed, degassed (2000 rpm and 5 min for each mode), and stirred for 24 h at 60 rpm in a ball mill. The nominal diameter of the micro-gripper tip was 300 μm, while the effective force could reach values of between 0.01 and 0.04 N and the tip displacement varied between 20 and 80 μm, respectively. Alblalaihid et al. [88] demonstrated the application of a sputter-coating process for the deposition of metallic layers on polymer components and validated their approach for the fabrication of a micro-gripper device (Figure 1C). They used a 3D projection micro-stereolithography (PMSL) system. The gripper was thermally actuated and the tip displacement, in this case, was in the range of 10–180 μm, depending on the applied potential.

### 3.2. Soft Robotics Applications

**Figure 2:**
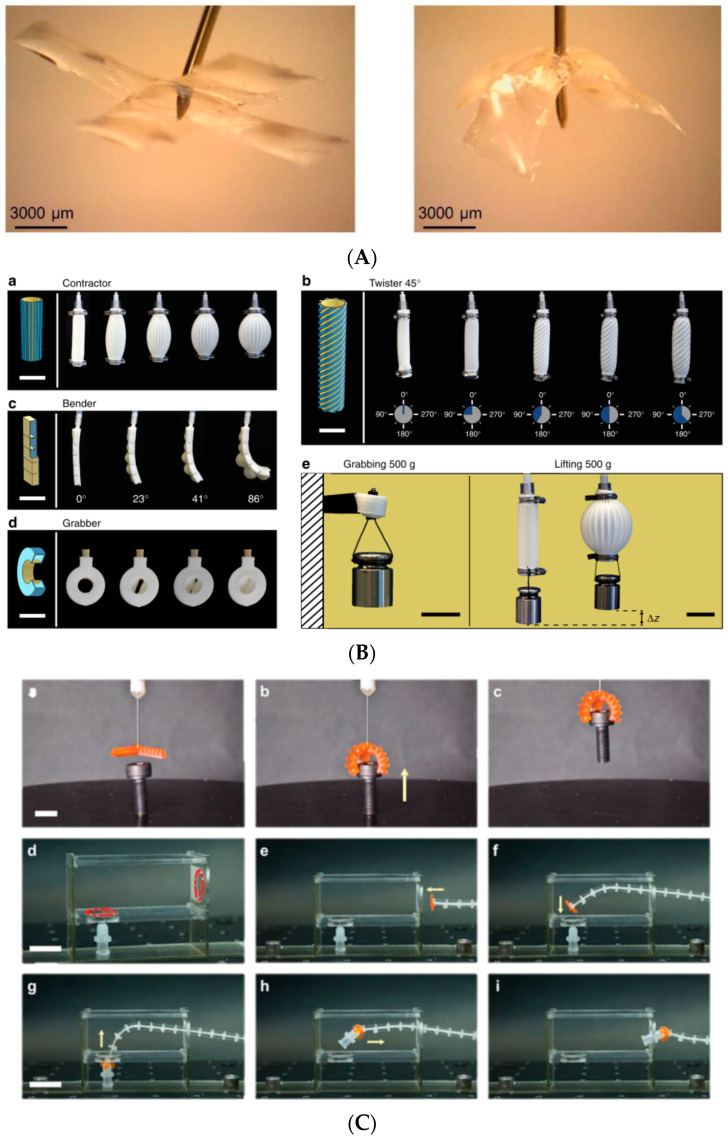
Examples of fabricated soft robotics: (**A**) Omnidirectional soft pneumatic actuator. Reprinted with permission from Ref. [84]. Copyright 2020 IOP Publishing. (**B**) Examples of soft actuators with programmed motion modes, fabricated from soft silicones. Reprinted with permission from Ref. [92]. Copyright 2018 Nature Communications, (**C**) Operation of the fabricated soft pneumatic gripper: the debris removal process within a confined space. Reprinted with permission from Ref. [95]. Copyright 2019 Wiley Online Library.

Besides micro-grippers, which, in most cases, maintain a rigid-type behavior during their operation, flexible actuators or soft robotics yield another application of microscale AM. Almeida et al. [89] designed an actuation mechanism for robotic micro-tweezers, based on a 3D-printed nylon flexure and a piezo-bimorph actuator, targeting the desired manipulation range from 100 µm to 1 mm. Bas et al. [90] designed miniature inflatable bending actuators, consisting of ultra-fine fibers (diameter of between 1 and 50 μm) and a soft elastomer matrix able to exhibit diverse movements. They used melt electro-writing (MEW) technology to create the prototypes (Figure 2B). Their actuators, with a length of 10–15 mm and an inner diameter of 1 mm, can reach their full range of motion within ~20 ms without exploiting snapping instabilities or material non-linearities. Joyee and Pan [91] fabricated a fully 3D-printed multi-material, multi-modal functional soft monolithic robot, composed of polymer and magnetic particle-polymer composites. The fabrication process was magnetic field-assisted projection stereolithography (M-PSL), capable of fabricating smart particle-polymer composites layer by layer. A photocurable flexible resin was used as the base material for 3D printing, while the magnetic nanoparticles (10 nm in nominal diameter) contained 60–80 wt % iron oxide. The maximum bending deformation was 5.2 mm on the z-axis and the maximum deflection in the xy plane was 146°. Schaffner et al. [92] reported a 3D-printing platform for the seamless digital fabrication of pneumatic silicone actuators, exhibiting programmable bioinspired architectures and motions with spatial resolutions in the range of 300 μm. They used viscoelastic silicone inks, resulting in elastomers with variable stiffness after polymerization. Sinatra et al. [93] introduced a novel fabrication strategy for nanofiber-reinforced soft micro-actuators with 30 μm feature sizes. The design and manufacturing of composite polydimethylsiloxane (PDMS)/nanofiber actuators using soft lithography and rotary jet spinning are described. Among the examined parameters were the lamina design and fiber orientation on the actuator curvature, mechanical properties, and pressurization range. Composite actuators displayed a 25.8% higher maximum pressure than pure PDMS devices. Furthermore, the best nanofiber-reinforced laminates tested were 2.3 times tougher than the control PDMS material, while maintaining comparable elongation. Xavier et al. [94] presented the design and direct 3D printing of novel omnidirectional soft pneumatic actuators using SLA (Figure 2A). They used an elastic resin and FDM with a soft thermoplastic polyurethane (TPU), achieving multimodal actuation including bending, extension, and contraction motions under positive, negative, or differential pressures. The printing time for a single actuator using the SLA method was 6 h and 40 min while the printing time using the FDM method was approximately 29 h and 20 min. In [95], Zhang et al. presented a generic process flow for the systematic and efficient tailoring of the material formulation and key processing parameters for the digital light processing-based 3D printing of miniature pneumatic actuators for soft robots. They printed various miniature pneumatic robots with an overall size of 2–15 mm and a feature size of 150–350 µm. They used a commercially available UV-curable elastomer, to which was added 30 wt % epoxy aliphatic acrylate (EAA), leading to a reduction in Young’s modulus and an increase in failure strain. All the specimens were post-cured for 10 min. Ge et al. [96] presented the design of a bottom-up digital light processing (DLP) 3D printer system (385 nm UV light source, 50 μm normal resolution) and the fabrication of multiple-size soft pneumatic actuators integrally, with fast speed and high precision. Their experiments demonstrated that the printer could print objects with features as small as 87.5 μm. They also presented the design and fabrication of a soft pneumatic gripper containing three micro pneumatic actuators with 0.4-mm-wide square air channels, as well as 0.2-mm-thick chamber walls.

### 3.3. Biomedical and Microfluidics Applications

Microscale AM has been efficiently used in biomedical engineering, including many microfluidic applications, which can also be treated as a separate category. The methods, potential, challenges, and limitations of microscale AM in biomedical engineering have been reported in recent review studies. The applications of 3D printing in the health and pharmaceutical sectors have been thoroughly investigated over the last few years and can be tracked in the following review papers [97,98,99]. According to the authors of [99], the applications can be divided into the following categories: 3D disease modeling, pharmaceutical products, organ printing, and patient-specific in situ implants. Other possible applications include drug-delivery devices [100,101,102], the fabrication of microneedles [103,104], microfluidic devices and biomedical micro-devices [105,106], and the fabrication of tissues [107,108].

**Figure 3:**
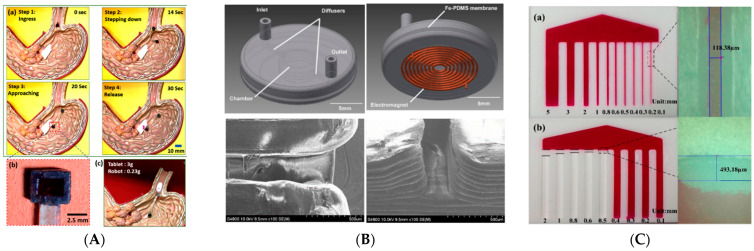

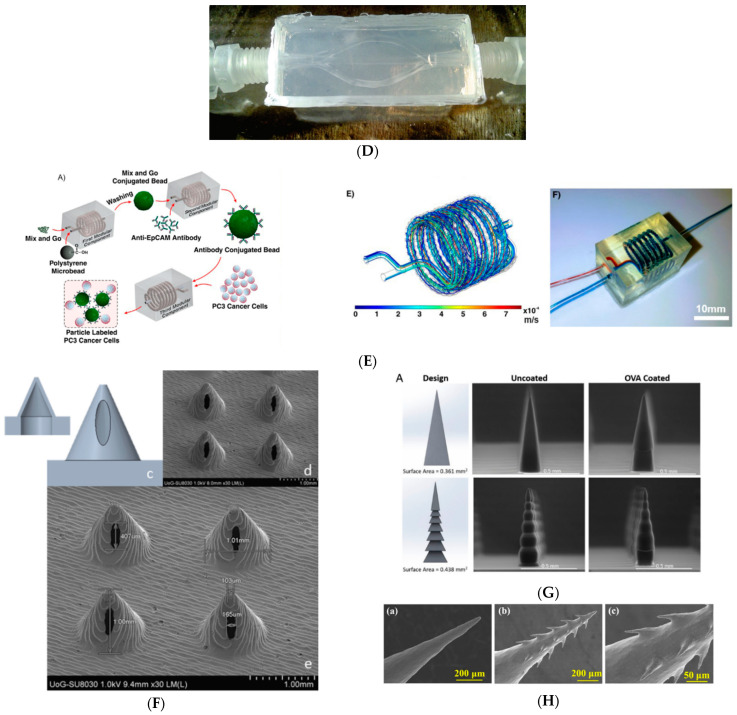
Examples of biomedical devices: drug delivery applications, microchannels, and microneedles: (**A**) Presentation of the operation of the multi-material soft robot. Movement of the actuator inside an anatomical stomach model with cancer tissue (target) and release of the drug at the targeted tumor location. Reprinted with permission from Ref. [109]. Copyright 2020 Elsevier. (**B**) Electromagnetically actuated microfluidic pump. Reprinted with permission from Ref. [113]. Copyright 2016 Elsevier. (**C**) Fabricated hydrophilic channels and hydrophobic chambers. Reprinted with permission from Ref. [122]. Copyright 2016 MDPI. (**D**) 3D printer head for additive manufacturing of sugar glass for tissue engineering applications. Reprinted with permission from Ref. [123]. Copyright 2017 Elsevier. (**E**) The design of microchannels for mixing: concentration distribution, streamlined simulations, and fabricated micromixer. Reprinted with permission from Ref. [111]. Copyright 2020 Elsevier. (**F**) CAD images of the microneedle designs and respective cross-sections, along with SEM images. Reprinted with permission from Ref. [116]. Copyright 2021 Elsevier. (**G**) Design and environmental scanning electron microscope (ESEM) images of printed microneedles, pyramidal, and faceted designs. Reprinted with permission from Ref. [118]. Copyright 2021 National Academy of Science (**H**) Micro barb features of printed microneedles with SEM images. Reprinted with permission from Ref. [119]. Copyright 2018 American Chemical Society.

Drug delivery applications incorporate design solutions characterized by microscale features where AM has been successfully incorporated. In [109], Joyee and Pan proposed the design of a 3D-printed soft robot capable of multimodal locomotion. Utilizing computer aided design and computer aided engineering (CAD-CAE) tools for the design, they printed the robot via a novel magnetic field-assisted projection stereolithography (M-PSL) technique (Figure 3A). This soft robot is capable of bi-directional bending in the *xy* plane and *z*-direction and consists of anterior and posterior legs that contain a drug. The maximum dimensions of the robot in width and height is 5 mm × 5.5 mm, while the drug is released from a 200 μm hole.

In the field of microfluidics, Coltelli et al. [110] combined microfluidics, AM, and electrostatic actuation to design artificial muscles capable of generating up to 33 Mpa stress and 10–20% strain. Their design consists of arrays of rectangular cavities arranged accordingly, filled with conducting material inside a bulk dielectric volume. They suggest that the microfluidic devices are AM-fabricated in such a way that the channels would form wiring when filled with conducting fluid, while the bulk core would serve as the dielectric and as the force-transfer medium. The non-flexed lateral size of the electrode plates was kept at 400 μm × 400 μm. The non-flexed plate thickness was kept at 100 μm for each plate and the non-flexed separation between paired plates within the same micro-capacitor was kept at 100 μm. The accuracy of the SLS printing was kept at 100 μm. In [111], the authors illustrated the direct fabrication of a 3D complex microchannel design using AM, for the continuous mixing of micro/nano-particles with biomolecules. The fabrication process was conducted using the DLP method. After the 3D printing stage, the part was removed and washed with IPA (70% ethanol and water), blow-dried with pressurized air, and, finally, cured under UV light for 120 s. The cross-section of the trapezoidal channel had a width of 600 μm and heights of 80 and 130 μm. Another example of a design of microfluidic MEMS was presented in [112], where the authors proposed a micro-extrusion 3D printing system that contained integrated pick-and-place functionality. The case study was the fabrication of microfluidic-based 3D MEMS (three-dimensional microelectromechanical systems) that contain orthogonal out-of-plane piezoelectric sensors and actuators, using additive manufacturing.

Miniature pumps are very critical components in the health sector. In [113], Thomas et al. fabricated a 3D-printed electromagnetically actuated microfluidic pump, capable of generating a 2.2 μL/min flow rate of biofluid (Figure 3B). An FDM process with 100 μm-layer resolution was used to deposit polylactic acid on a plastic filament. Taylor et al. [114] fabricated a multi-material miniature diaphragm pump for the creation and maintenance of a low vacuum from atmospheric conditions, using PolyJet printing. The output surface was assessed in terms of roughness, giving values of R_a_ in the order of some microns (~2–3 μm), while the R_z_ values were close to layer thickness (~16–18 μm), which was considered acceptable. The stroke of the pump was 2.5 μm. In [115], a low-cost (~$120), open-source peristaltic pump was constructed with a combination of 3D-printed parts and common hardware. The pump was capable of producing flow rates of up to 1.6 mL min^−1^.

In the field of microneedles (MNs), Economidou et al. [116] fabricated a hollow MN MEMS system for controlled transdermal drug delivery. They fabricated hollow cone-shaped MNs with a base diameter of 1000 µm, a tip diameter of 100 µm, and a height of 1000 µm using SLA and, afterward, integrated the MNs onto the MEMS. The hollow cones featured a wall thickness of 100 µm and the internal bores had a diameter of 800 µm at the cone base. The MNs were fabricated using an SLA 3D printer, followed by curing for 60 min under 40 ℃ UV radiation. The authors observed smooth surfaces on the MNs (no “stair-stepping” effect), as an outcome of the printing method they selected. In [117], the authors provided the capabilities of FDM low-budget printers (using PLA printing material) to print the non-transparent and closed internal microfeatures of in-plane linear, curved, and spiral microchannels with a diameter of less than 0.5 mm (i.e., linear, curved, and spiral channel profiles) and varying cross-sections. The surface roughness of each microchannel configuration was measured and was found to be in the order of some microns (~0.5–3 μm). In addition, each configuration was tested in terms of leakage flow. Caudill et al. [118] designed and printed microneedle arrays utilizing a three-dimensional (3D)-printing technique called continuous liquid interface production (CLIP). Besides pyramidal MNs, the design involved faceted MNs with horizontal grooves, leading to an increase in surface area and, thus, better vaccination properties. The MNs were 700 μm in height and 500 μm in width and were printed in a 10 × 10 array on a 10 mm × 10 mm patch for vaccine delivery. Chen et al. [119] proposed a novel 3D AM method, known as magnetorheological drawing lithography (MRDL), to efficiently fabricate bio-inspired MNs imitating the honeybee’s stinger. With the assistance of an external magnetic field, a parent MN (20 μm tip width) was directly drawn on the pillar tip, and tilted micro barbs (5 μm tip width) were subsequently formed on the four sides of the parent MN. The fabrication process of the parent MN was conducted by means of insertion and, afterward, the removal of a copper pillar inside a pool filled with curable magneto-rheological fluid (CMRF) under an external magnetic field. Micro barbs were formed later, on the curved surface of the parent MN. Compared with a barbless microneedle, the micro-structured barbs enabled the bio-inspired microneedle to be easily inserted into the skin, with difficult removal. In [120], the authors used a commercially available stereolithographic 3D printing, which was assessed regarding its microscale fabrication properties, in order to fabricate sharp MNs (12 × 12 array, in total, 144; 30 min per patch) with a tip radius of approximately 15 μm. In another study [121], a microneedle mold fabrication technique using a low-cost desktop SLA 3D printer was presented, and the fabrication of needles with high-aspect ratios and tip radii of 20–40 µm took place.

Examples of different biomedical devices produced via AM are presented in Figure 3.

## 4. Design Considerations

Additive manufacturing allows the production of parts with almost no geometry restrictions, even at a microscale. This manufacturing method can produce freeform, topology-optimized geometries (structures, arrays, patterns, small assemblies, micro-machines, etc.), controlling the micro-structure of the component’s material, and implementing the use of lattice structures, trusses, and multi-material fabrication. This way the engineer is able to “design” the structure of the material in macro- and even microscale, controlling the material properties based on the functionality of the component.

One may consider that the design for AM at the microscale deviates from the design protocols and approaches at the macroscale. From a general perspective, design at the macroscale is based on the principle of integrating parts into assemblies, while the interconnection or mating is achieved via the use of fundamental machine elements and machine-design methods, such as screws, tight fits, weldments, etc. Therefore, the functionality and the operational precision of a complex assembly are limited by the number and the attributes of the elements comprising it, since the rule of thumb is that as the number of the parts increases, precision drops. AM can be seen as an approach for creating more complex forms of parts-like assemblies, which is critical for applications at the microscale since many of the available micromachining techniques are mere extensions of their macroscale equivalents. This might attract questions and ambiguity regarding the appropriateness of the physics and the operational principle of the method due to the scale aspect; nevertheless, the alternative approach of fabricating micro machines with increased functionality and fewer but more complex parts is intriguing. For instance, multiple degrees of freedom in robotic arms can be achieved via the use of joints that are translated into several components (motors, bearings, screws, etc.) but in small-scale soft robotics, a single monolithic part made of soft material is able to derive controllable motions. Thus, AM is an alternative approach to designing at microscale, with an entirely different basis.

**Figure 4:**
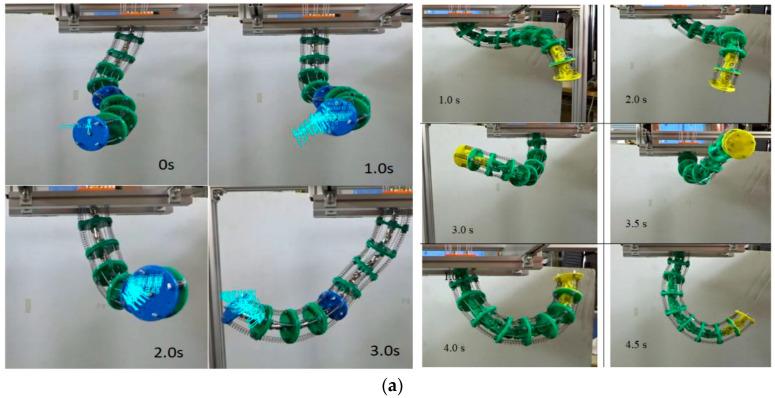

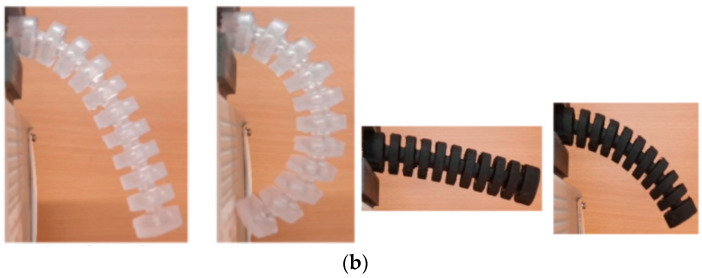
Joints in macro and microscale: (**a**) Testing of the wire-driven robot arm. Reprinted with permission from Ref. [124]. Copyright 2019 MDPI. (**b**) Experiments on bending deformation due to applied pressure from an omnidirectional soft pneumatic actuator. Reprinted with permission from Ref [94]. Copyrights 2021 Elsevier.

In Figure 4, joints in the macro- and microscales are presented. Specifically, Figure 4a shows the testing of a wire-driven continuum robot arm [124], while Figure 4b experiments with bending deformation due to the applied pressure of an omnidirectional soft pneumatic actuator [94].

In order to fully utilize the capabilities of AM and reduce design and fabrication defects, it is critical to quantify each process’s parameters, limitations, and repeatability. For this purpose, numerous analytical methods were developed, along with many computational tools. Despite these methods and tools, experimental evaluation remains crucial for the optimization of AM processes.

### 4.1. Comparative Advantages and AM Limitations

**Figure 5:**
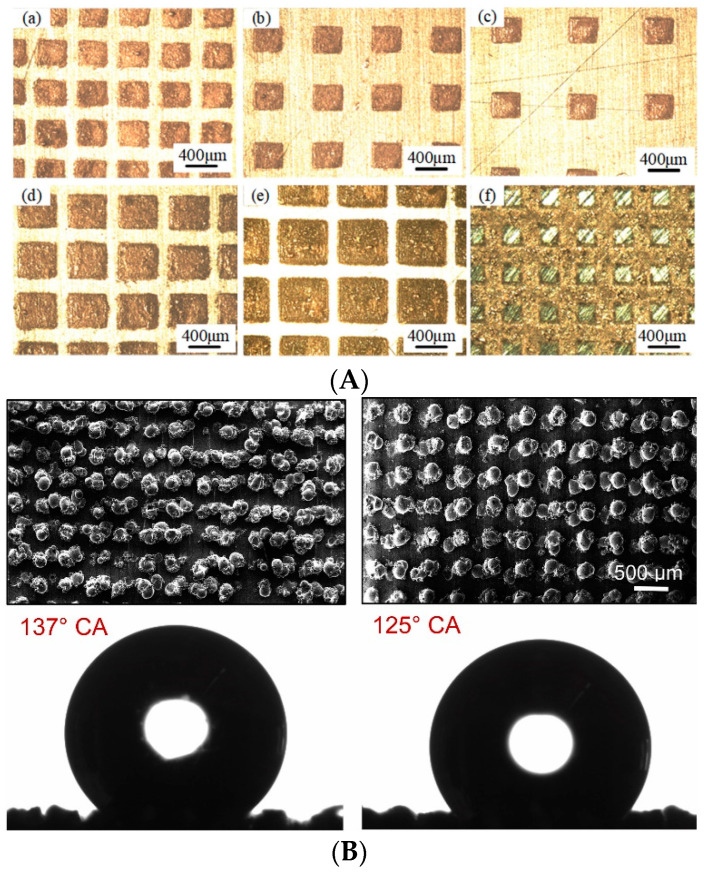
Examples of AM textures. (**A**) Various textures made of ink-printed copper nanoparticles. Reprinted with permission from Ref. [149]. Copyrights 2019 Elsevier. (**B**) Two cases of pillar array surfaces and the measured contact angle. Reprinted with permission from Ref. [150]. Copyrights 2021 Elsevier.

AM enables the fabrication of complex, freeform, and smart structures [125]. Among their other unique capabilities, the use of lattice structures for the topology optimization of structures, and the design of lightweight components is possible. Nature-inspired design for the mimetics of complex nature layouts is also feasible via AM. The aim of these approaches is usually to yield controllable mechanical properties that can be tuned according to the requirements of the application. Lightweight structures, energy absorption, the fabrication of nature-inspired micro-patterns, and modeling and simulation techniques are important state-of-the-art aspects in the field of cellular micro-lattice architectures. Moreover, AM enables the control of the microstructure of the material, allowing the design of desired component properties [126]. In this way, AM could be used for the on-demand production of metamaterials. Metamaterials are ordered composites that have material properties not usually found in nature. The use of auxetic and custom infill patterns that are directly optimized to transfer energy absorption properties or dumping capabilities to AM components leads to unique smart materials and highly efficient components. Multi-material fabrication poses new challenges in the design and fabrication of smart components [26]. Recent advancements in the field of design, modeling and simulation, fabrication, and testing of lattice structures can be found in the following review papers [127,128,129,130,131,132,133,134,135,136]. Deriving the effective properties of additively manufactured micro-lattice structures is an important tool in the hands of designers for performing fast simulations at a low computational cost [126,137,138,139]. Souza et al. [139] derived a closed-form analytical solution of lattice structures fabricated by selective laser melting, using beam models. Athanasiadis et al. [140], in work based on fracture mechanics theory, investigated the potential of lattice structures to replace adhesives in sandwich-type structures, using both analytical and FEA calculations. Kenel et al. [141], using 3D ink extrusion, fabricated CoCrFeNi micro-lattices with strut diameters as narrow as 100 μm, and tested their compression and tension properties at ambient and cryogenic temperatures. Boulvert et al. [142] tested the acoustic behavior of 3D-printed micro-lattices in order to extract conclusions about the defects of FDM. The size of their samples was in the order of 200 μm, and defects included the presence of micro-grooves on the lattices’ surfaces in the order of 10 μm. Studies regarding the defects of lattices were also conducted in [143,144,145,146]. In [147], McGregor et al. conducted a statistical study in order to assess the geometric quality of 2D and 3D micro-lattice structures. At the same time, as lattice structures outstripped bulk cores in many technological applications, the introduction of artificial, additively manufactured, textures come forward as an efficient tool for the fabrication of surfaces. Additive texturing is a state-of-the-art approach for fabricating surfaces with superior tribological, wetting, and wear characteristics [148]. In [149], Wang et al. used the selective laser melting of ink-printed copper nanoparticles (SLM-IP Cu NPs) in order to fabricate a friction-reduced surface for operating in mixed-lubrication conditions (Figure 5A). The patterns investigated were concave and convex, squared and fully sintered Cu film; the height of the features was 20 μm. Mekhie et al. [150] printed metallic hierarchical micro-features (pillars, channels, etc.) using selective laser melting (Figure 5B) for the wetting control, achieving hydrophobic surfaces with a contact angle greater than 140°.

Consequently, AM is a rapidly evolving manufacturing process, with huge potential to revolutionize the fabrication of functional components. Although it is considered a method with unlimited capabilities, limitations do exist. These limitations take into consideration CAD digitization, process parameter optimization and the effect on material properties, the current capabilities of AM technologies, and the lifecycle of AM components, as well as metrology and quality control challenges [151]. 

Among the most popular technologies, such as SLS, stereolithography, and FDM, the need to expand the materials used for microscale AM is a major challenge. The adaptation of non-metallic materials, such as ceramics, polyamides, and composite powder-enriched resins, is necessary for the improvement of component functionality, as well as the expansion of the applications for which AM is used [26]. Another important limitation for most of the aforementioned processes is the fabrication of hollow, closed structures as it is difficult to remove excess material without invasive post-processing. Dimensional and geometrical deviations, linked with thermal history, heat-affected zones (HAZ), and material phase changes, impose great restrictions on AM processes when it comes to functional component fabrication. LOM, for example, is known to undergo severe shrinkage by as much as 18% in some cases. Especially in sintering, the feature size of the component is limited either by the particle-size limitations of the powders used or the technology’s laser focus [152,153]. Moreover, increased surface roughness is also connected with powder size and HAZ during fabrication, further restricting the quality of AM components [154,155]. One of the most frequent defects in sintering processes, leading to poor mechanical properties and a decreased life cycle, is porosity. Being affected by both environmental and process parameters, along with thermal and oxidation effects, porosity is one of the most important constraints for both macro- and microscale AM [26,46].

When it comes to stereolithography, minimum layer thickness, as well as improved surface roughness, are challenging. Both are limited by the physical properties of the resins used. Surface tension and the viscosity of the resin are the limiting factors for layer thickness, and also affect the surface quality and post-processing needed to clean up the final component by removing all excess resin.

### 4.2. Selection of Processes and Materials

As previously described, AM consists of many different technologies (processes), each of them with unique capabilities and limitations. Every different technology uses specific materials or groups of materials. Thus, a critical point of the design process is the selection of a particular AM technology to utilize its advantages and obtain the best manufacturing quality, as well as achieve the optimal material based on the functionality of the fabricated component. For example, micro-stereolithography is a high-resolution method that uses photocurable resins capable of producing complicated components in large manufacturing volumes. Moreover, materials such as ceramics, metals (WC, Co, Al, Cu), and hydrogels can be used [156,157,158,159]. Micro-laser sintering is a powder-based method with a wide variety of materials, isotropic properties, and without the need for support structure during component fabrication. Among the most popular materials of MLS are 316 L stainless steel and a variety of metals. However, the technique requires post-processing and the components may suffer from porosity [160]. FDM is one of the most popular macroscale technologies that can also fabricate components in microscale. Besides thermoplastics, which are widely used, biomaterials are also available, making it possible to create medical and biological parts. Nevertheless, it is limited to low fabrication volumes, with high temperatures and poor repeatability [158,161]. Laminated object manufacturing (LOM), one of the oldest AM processes, uses metals such as 316 L stainless steel, zirconia, and ceramics for the fabrication of fully dense and high-mechanical-strength components. Its resolution, however, is limited to 80 μm; it lacks dimensional accuracy due to high shrinkage (12–18%), and the post-processing of parts is required [161,162]. The popular, 2D-based, Inkjet printing process uses a wide variety of materials and many biomaterials, making it ideal for biomedical applications and with fair repeatability, but the need for support is essential [26]. The other 2D-based method, FIBDW, uses mostly metals for the fabrication of high-resolution components but remains a slow method with poor repeatability [162,163]. Finally, one of the most widely used AM methods for micro-part fabrication, EFAB, is suitable for the high-resolution manufacturing of complex parts and assemblies, such as medical devices. Nevertheless, it has dimensional limitations and post-processing is necessary and sometimes even difficult [72,164,165].

### 4.3. Design Considerations

**Figure 6:**
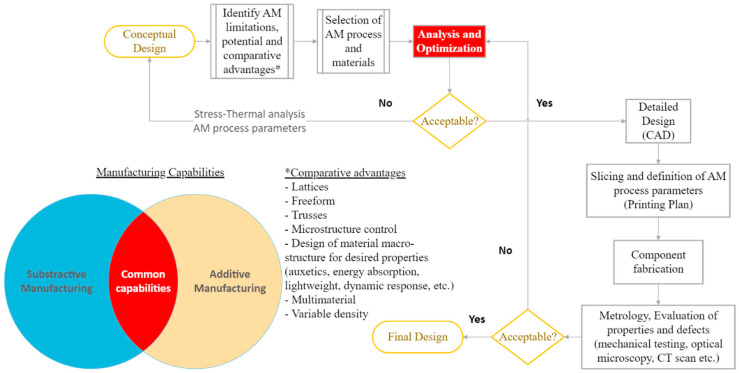
Design for Additive Manufacturing process.

As discussed thoroughly in the previous paragraphs, AM, despite being a revolutionizing fabrication method with unique capabilities, still has limitations and restrictions when it comes to functional component manufacturing. The proposed actions during the process of design for AM are presented in Figure 6.

The designer, on the one hand, needs to exploit all the comparative advantages of AM methods, such as freeform fabrication, the use of lattices and trusses, the ability to control the microstructure, and the use of metamaterials with advanced properties. On the other hand, the designer has to consider the limitations and weaknesses of each fabrication method. Therefore, the design process begins with the conceptual design of the component, based on functional and geometrical criteria. Then, the designer must select the most suitable AM method by taking into consideration the material, process limitations, and fabrication possibilities. After the material and process are selected, an optimization procedure is carried out, in order to minimize defects and increase repeatability and productivity. This optimization procedure involves coupled stress-thermal analysis to exploit the effects of the AM process on the final material properties (residual thermal stresses, density, material anisotropy, strength, fatigue life, etc.). Based on the large number of different fabrication methods and the limited computational tools that can accurately predict stress-thermal behavior during fabrication, many experimental methods are also popular for the optimization and evaluation procedures. Last, but not least, the optimization procedure also includes another critical aspect, the process parameter selection. Recent advances in the technology of AM and software development dedicated to AM fabrication allow the control of a variety of critical parameters for AM fabrication. This software uses dedicated algorithms that propose an optimized set of parameters and allow the user to manually intervene and control them. Finally, in the same way as in traditional subtractive manufacturing methods, metrological evaluation is necessary to ensure the quality of the fabricated component. On the microscale, the use of conventional metrological equipment is impossible. Therefore, other methods and procedures had to be created or adapted to cover the gap. Some of them are presented in the next paragraph.

### 4.4. Metrological and Performance Validation of AM Structures at the Microscale

The experimental evaluation of components produced with AM is critical for the optimization of the manufacturing process and the minimization of defects and deviations from nominal geometry. The micro-detail (mD) measurement of complex parts remains an open field of investigation. It is critical to experimentally establish the minimum dimensional and geometric limits to produce parts with mDs. These limits are highly dependent on the AM process, technology, and materials used [166,167].

One way of doing that is based on mathematical formulations, using voxelization (a process where the geometry of the part is represented with volumetric unitary elements). The minimum feature size and obtainable tolerances of additive manufacturing processes are linked to the smallest volumetric elements (voxels) that can be created. These models can predict the behavior of the parts during fabrication. A test sample can then be used to evaluate the dimensional variation [168,169].

Dimensional restrictions in the microscale make the use of conventional metrological equipment impossible. One very popular alternative for the evaluation of AM components in microscale is X-ray computed tomography (XCT). The use of XCT for testing and analyzing AM components was established in the past few decades and is becoming extremely popular for dimensional evaluation, as well as structural integrity, density, and porosity analyses [170,171].

Another popular evaluation method is optical microscopy. This method can be non-destructive for the examination of surface quality and external geometrical characteristics, but it can also be used as a destructive method to obtain information about the internal porosities of a component [172,173].

## 5. Conclusions

This article presents advancements in the field of additive manufacturing at the microscale from a design perspective. The main ambition of this study is to highlight the critical aspects of the design process by combining generalized results, mainly regarding the effect of fabrication technologies and the use of materials. Even though microscale AM technologies have been extensively discussed in other recent studies, here, we conclude that the fabrication of microscale components using AM may be employed either by utilizing existing meso- or macroscale techniques or by developing new, dedicated methods at the micro- and nanoscales. Furthermore, this article collects recently presented applications of microscale AM in the field of the fabrication of MEMs, actuators, and soft robotics, as well as in the biomedical sector. The design process presented begins by considering the comparative advantages and limitations of microscale AM. Its advantages include freeform fabrication, the use of lattices and trusses, the ability to control the microstructure, and the use of metamaterials with advanced properties. The limitations are mainly associated with CAD digitization, the effects of fabrication process parameters on material properties, which must be assessed, and the lifecycle of AM components, as well as the current lack of metrology and quality control protocols. The design procedure proceeds with the selection of appropriate materials and processes, analysis and optimization using CAE, and detailed design, then concludes with fabrication and the post-evaluation.
